# Assessing the Labeling Information on Drugs Associated With Suicide Risk: Systematic Review

**DOI:** 10.2196/49755

**Published:** 2024-01-30

**Authors:** Soo Min Jeon, HyunJoo Lim, Hyo-bin Cheon, Juhee Ryu, Jin-Won Kwon

**Affiliations:** 1 Jeju Research Institute of Pharmaceutical Sciences College of Pharmacy Jeju National University Jeju Republic of Korea; 2 College of Pharmacy Kyungpook National University Daegu Republic of Korea; 3 Research Institute of Pharmaceutical Sciences College of Pharmacy Kyungpook National University Daegu Republic of Korea; 4 BK21 FOUR Community-Based Intelligent Novel Drug Discovery Education Unit College of Pharmacy and Research Institute of Pharmaceutical Sciences Daegu Republic of Korea

**Keywords:** suicide, adverse drug events, review, drug, mental health, systematic review, drug induced suicide, drug reaction, substance use, suicidal, medication, suicide symptoms, suicidal risk, drugs, adverse drug event

## Abstract

**Background:**

Drug-induced suicide (DIS) is a severe adverse drug reaction (ADR). Although clinical trials have provided evidence on DIS, limited investigations have been performed on rare ADRs, such as suicide.

**Objective:**

We aimed to systematically review case reports on DIS to provide evidence-based drug information.

**Methods:**

We searched PubMed to obtain case reports regarding DIS published until July 2021. Cases resulting from drugs that are no longer used or are nonapproved, substance use, and suicidal intentions were excluded. The quality of each case report was assessed using the CASE (Case Reports) checklist. We extracted data regarding demographics, medication history, suicide symptoms, and symptom improvement and evaluated the causality of DIS using the Naranjo score. Furthermore, to identify the potential suicidal risk of the unknown drugs, we compared the results of the causality assessment with those of the approved drug labels.

**Results:**

In 83 articles, we identified 152 cases involving 61 drugs. Antidepressants were reported as the most frequent causative drugs of DIS followed by immunostimulants. The causality assessment revealed 61 cases having possible, 89 cases having probable, and 2 cases having definite relationships with DIS. For approximately 85% of suspected drugs, the risk of suicidal ADRs was indicated on the approved label; however, the approved labels for 9 drugs, including lumacaftor/ivacaftor, doxycycline, clozapine, dextromethorphan, adalimumab, infliximab, piroxicam, paclitaxel, and formoterol, did not provide information about these risks.

**Conclusions:**

We found several case reports involving drugs without suicide risk information on the drug label. Our findings might provide valuable insights into drugs that may cause suicidal ADRs.

## Introduction

Suicide is a serious global public health problem. According to a 2019 report from the World Health Organization, more than 700,000 people die annually by suicide, implying that 1 person dies by suicide every 40 seconds [1]. Suicide results in fatal outcomes, enormous emotional distress for families, and a significant economic burden on society [2]. Therefore, much attention is required to reduce the burden of suicide. Suicide is associated with multiple factors, such as sex, age, history of suicide attempts, familial history of suicide, alcohol use, psychiatric disorders, and physical disorders. Among the identified risk factors, psychiatric and physical health disorders are the main controllable factors that prevent suicidal adverse drug reactions (ADRs). However, therapeutic drugs for these disorders have also been reported as a cause of suicide.

Psychiatric drugs, such as antidepressants, anxiolytic drugs, and antipsychotics, are representative drugs that have potential adverse effects, such as suicidal behavior. For instance, Stone et al [3] have conducted a meta-analysis involving 372 randomized controlled trials of antidepressants and reported that the odds ratio for suicidal behavior and ideation among patients aged younger than 25 years was 2.30 (95% CI 1.04-5.09). Moreover, several studies have demonstrated that some commonly prescribed drugs, such as hormonal contraceptives and beta-blockers, may induce suicidal ADRs [4,5]. According to a study by Qato et al [6], labels of 103 drugs indicated suicidal symptoms as side effects and the rate of administration of these drugs increased by approximately 1.09-1.35 folds from 2005-2006 to 2013-2014. Moreover, they revealed that patients who took these medications had approximately 5.3% greater concurrent symptoms of these adverse events than those who did not.

Owing to the growing awareness regarding drug-induced suicide (DIS), several studies have investigated the association between specified drugs and suicide [7-15]. Most of these studies involved case-control and cohort study designs and disproportionality analysis of pharmacovigilance databases. However, investigating the overall suicidal risk of all available drugs is challenging. Furthermore, the databases used in observational studies may not fully account for detailed clinical presentations, symptoms, and all other relevant factors contributing to suicide risk, such as preexisting mental health conditions, social support, and family history. Therefore, more comprehensive research is necessary to fully understand the potential association between all available drugs and DIS, considering individual patient factors and drug interactions.

In pharmacovigilance, case reports are crucial in identifying new safety signals and alerting physicians regarding the potential rare ADRs [16]. Case reports provide detailed clinical presentations and increase awareness regarding drug safety, although they are limited in establishing causality evidence. Thus, this study aimed to conduct a systematic review of published case reports on DIS associated with all available drugs. We further assessed the causality of DIS in each case and compared it with drug labeling information to establish new safety signals of DIS.

## Methods

### Overview

We conducted a systematic review of case reports on DIS published until July 2021 according to the PRISMA (Preferred Reporting Items for Systematic Reviews and Meta-Analyses) reporting guidelines (Multimedia Appendix 1) [17]. The literature search was performed on PubMed based on a combination of the following keywords—“suicide” and “drugs”—using Medical Subject Headings and text words. Two independent authors validated the search strategy, using the detailed search terms presented in Textbox S1 in Multimedia Appendix 2. For drug keywords, we used the active ingredient and generic name of the drug to obtain as many case reports as possible. The active ingredients or generic names of drugs were searched on “DrugBank.” We limited the article type and language to case reports and English, respectively.

### Study Selection

In this systematic review, we included case reports on suicidal behavior after administering the prescribed medications. We defined suicidal ideation and behavior as self-harm, suicidal ideation, and completed suicide. The exclusion criteria were (1) case reports on drugs that were no longer used or were nonapproved, (2) case reports on patients who took drugs for suicidal purposes, and (3) case reports without any mention of suicidal ADRs (eg, suicide genes). There were no restrictions on patient conditions, such as age, sex, psychiatric status, and outpatient or inpatient treatment.

Two authors with a background in pharmacology were independently involved in the process of article selection. They screened the titles and abstracts of all searched articles that met our eligibility criteria. If no consensus was reached between the 2 authors, a third independent author was solicited to make the final decision.

### Data Extraction

We extracted data from relevant case reports regarding patient demographics (ie, age, sex, psychiatric status, and socioeconomic status), medication history (ie, ADR, drug dosing, time to event, concomitant medication, and laboratory test), symptoms, improvement in symptoms after dose reduction or drug discontinuation, and causality assessment results.

### Data Synthesis and Analysis

For all cases of DIS, we summarized patients’ demographics, including age, sex, and underlying psychiatric status. The demographics of cases were presented as the frequencies and percentages. Age was categorized into 3 groups (<19, 19-59, and 60≤ years). Data were presented according to the classes of suspected drugs involved in DIS cases. The drug classes were classified according to the anatomical therapeutic chemical (ATC) code [18]. The third level of the ATC (ie, the chemical, pharmacological, or therapeutic subgroup) was mainly used to categorize drugs; some drugs were classified based on the second level of the ATC (ie, the pharmacological or therapeutic subgroup). Detailed code classification is presented in Table S1 in Multimedia Appendix 2.

Furthermore, we assessed the probability of drug administration that leads to DIS using the Naranjo ADR score [19], which has been widely used as a tool for causality assessment in case reports. This scale included 10 simple and clarified questions, and the answer to each question is categorized into 3 options “Yes,” “No,” and “Unknown.” This scale can consider the missing values when evaluating the causality. The calculated score was classified into 4 levels, as follows: doubtful (≤0), possible (1-4), probable (5-8), and definite (≥9). As some questions in the original version of the Naranjo algorithm were not detailed enough to assess causality, we defined and used detailed criteria (see Multimedia Appendix 3). Although few articles had already included information regarding the Naranjo score as a causality assessment, we reevaluated this score for all articles. The quality of the selected reports was evaluated using the CARE (Case Reports) guidelines [20].

### Investigation of Potential Drugs for DIS Risk

We compared the causality assessment results with corresponding label information to identify drugs with the potential risk of suicide. In this analysis, we used the average Naranjo score for each drug as a representative value of our causality assessment. We reviewed the label information for all suspected drugs using the Food and Drug Administration (FDA)–approved label and Micromedex and classified these drugs into 3 groups, as shown in Textbox 1 (high-, intermediate-, and low-level evidence). Drugs were considered “high-level evidence” if the label information indicated a direct expression of “suicide” (ie, suicide, suicide attempts, suicidal ideation, suicidal thoughts, suicidal behavior, suicidality, or suicide risk). Drugs were considered “intermediate-level evidence” if the label information did not directly indicate suicide but specified psychiatric disorders often associated with suicide-related symptoms (ie, depression, anxiety, delirium, hallucinations, and psychotic behavior). The remaining drugs were considered “low-level evidence.” In our case reports, if the suspected drugs associated with DIS were classified as low-level evidence according to the label information, we considered them as potentially unrecognized drugs for DIS.

**Current evidence levels for reported drugs associated with DIS based on the approved labeling information.**

High: Drugs with information that includes a direct expression of “suicide,” such as suicide, suicide attempts, suicidal ideation, suicidal thoughts, suicidal behavior, suicidality, and suicide risk.

Intermediate: Drugs with information regarding psychiatric disorders associated with suicide-related symptoms, such as depression, anxiety, delirium, hallucinations, and psychotic behavior.

Low: Drugs without any adverse drug reaction information for suicide and suicide-related symptoms.

## Results

**Figure 1:**
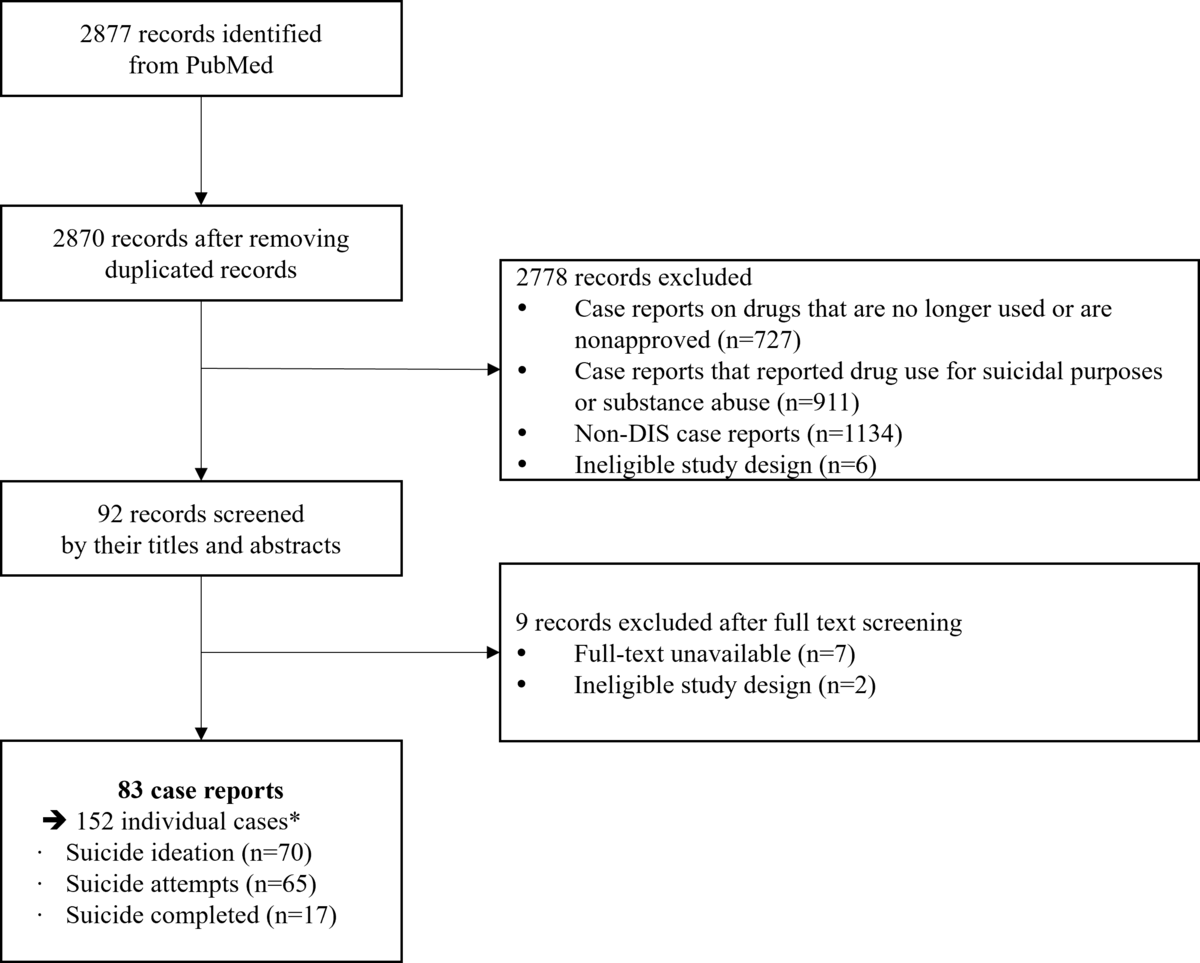
Flowchart of the DIS cases selection process. DIS: drug-induced suicide. *To consider thorough medications, we recognized 7 patients linked to multiple drugs; each case was treated individually, totaling 152 cases for detailed analysis; the number of patients who were duplicated because the cases were associated with more than 2 individual drugs.

We identified 2877 articles from the PubMed database and removed 7 duplicated records. After reviewing the titles and abstracts of these articles for eligibility, we excluded 2778 articles that met the exclusion criteria. Overall, 9 of the remaining 92 articles were excluded because they did not contain full text (n=7) or had ineligible study designs (n=2). Finally, we selected 83 articles that involved 152 individual drug-suicide relationships (Figure 1). In total, there were 70, 65, and 17 cases of suicidal ideation, suicide attempts, and completed suicide, respectively. The studies had total scores ranging from 17/30 to 24/30 according to the CARE checklist (Multimedia Appendix 4).

In these case reports, 61 individual drugs were identified as drugs associated with DIS and were grouped into 31 classes. Table 1 summarizes the demographics and characteristics of DIS cases according to the class of the suspected drugs. There were 26 cases of DIS related to antidepressants, which was the most frequently reported drug class. For antidepressants, most patients were aged between 19 and 59 years (n=16) followed by the age group of 2-18 years (n=8). A psychiatric diagnosis was made in all patients, except for 1; and 6 patients had a history of suicide attempts. In 25 patients (with a mean age of 37.8 years), immunostimulants were identified as the causative drug for DIS. A total of 7 patients showed a personal or family history of psychiatric disorders or suicide attempts. Antiepileptics were identified as the causative drugs for DIS in 15 patients, of whom 8 had a history of psychiatric disorders. Furthermore, 8 patients associated with anabolic steroids were all male and aged between 19 and 59 years (mean age of 26.5 years). All 8 patients associated with psychostimulants were aged 2-18 years (mean age 10.3 years; range 9-13 years), which was the youngest among all the drug classes.

**Table 1:** Demographics of suspected drug for suicide by drug classes.

	N^a^	Sex^b^			Age (years)^b^					History of psychiatric status^b^			
	(N/152), n (%)	Male, n (%)	Female, n (%)	Mean (range)		<19, n (%)	19-60, n (%)	60≤, n (%)	Psychiatric diagnosis, n (%)		Substance abuse, n (%)	Suicide attempts, n (%)	Family history, n (%)
Antidepressants	26 (17.0)	9 (34.6)	17 (65.4)	29.1 (10-62)		8 (30.8)	16 (61.5)	2 (7.7)	25 (96.2)		22 (84.6)	6 (23.1)	3 (11.5)
Immunostimulants	25 (16.3)	12 (48.0)	13 (52.0)	37.8 (19-62)		0 (0.0)	24 (96.0)	1 (4.0)	4 (16.0)		6 (24.0)	3 (12.0)	3 (12.0)
Antiepileptics	15 (9.8)	10 (66.7)	5 (33.3)	35.1 (10-66)		2 (13.3)	12 (80.0)	1 (6.7)	8 (53.3)		9 (60.0)	4 (26.7)	1 (6.7)
Anabolic steroids	8 (5.2)	8 (100.0)	0 (0.0)	26.5 (21-34)		0 (0.0)	8 (100.0)	0 (0.0)	7 (87.5)		7 (87.5)	0 (0.0)	4 (50.0)
Psychostimulants	8 (5.2)	6 (75.0)	2 (25.0)	10.3 (9-13)		8 (100.0)	0 (0.0)	0 (0.0)	8 (100.0)		8 (100.0)	0 (0.0)	4 (50.0)
Drugs used in addictive disorders	6 (3.9)	2 (33.3)	4 (66.7)	35.5 (19-67)		0 (0.0)	6 (100.0)	0 (0.0)	6 (100.0)		6 (100.0)	0 (0.0)	0 (0.0)
Antiacne preparations	6 (3.9)	5 (83.3)	1 (16.7)	44.8 (38-58)		1 (16.7)	4 (66.7)	0 (0.0)	4 (66.7)		5 (83.3)	0 (0.0)	0 (0.0)
AI^c^ and AR^d^ products, nonsteroids	5 (3.3)	3 (60.0)	2 (40.0)	19.2 (17-20)		0 (0.0)	5 (100.0)	0 (0.0)	4 (80.0)		4 (80.0)	0 (0.0)	1 (20.0)
Antipsychotics	5 (3.3)	4 (80.0)	1 (20.0)	47.6 (37-57)		0 (0.0)	4 (80.0)	1 (20.0)	5 (100.0)		5 (100.0)	0 (0.0)	0 (0.0)
Respiratory system products	5 (3.3)	0 (0.0)	5 (100.0)	15.0 (12-17)		5 (100.0)	0 (0.0)	0 (0.0)	3 (60.0)		3 (60.0)	0 (0.0)	0 (0.0)
Antibiotics	4 (2.6)	3 (75.0)	1 (25.0)	30.8 (18-53)		1 (25.0)	3 (75.0)	0 (0.0)	1 (25.0)		1 (25.0)	0 (0.0)	2 (50.0)
Antimycobacterial	4 (2.6)	4 (100.0)	0 (0.0)	35.5 (20-55)		0 (0.0)	4 (100.0)	0 (0.0)	0 (0.0)		2 (50.0)	1 (25.0)	1 (25.0)
Antivirals	4 (2.6)	3 (75.0)	1 (25.0)	30.0 (13-47)		1 (25.0)	3 (75.0)	0 (0.0)	2 (50.0)		3 (75.0)	1 (25.0)	2 (50.0)
Immunosuppressants	4 (2.6)	2 (50.0)	2 (50.0)	46.0 (32-56)		0 (0.0)	4 (100.0)	0 (0.0)	1 (25.0)		1 (25.0)	1 (25.0)	0 (0.0)
Analgesics and antipyretics	3 (2.0)	1 (33.3)	2 (66.7)	53.3 (39-66)		0 (0.0)	2 (66.7)	1 (33.3)	3 (100.0)		3 (100.0)	1 (33.3)	0 (0.0)
Anesthetics	3 (2.0)	1 (33.3)	2 (66.7)	39.3 (25-64)		0 (0.0)	2 (66.7)	1 (33.3)	2 (66.7)		3 (100.0)	1 (33.3)	1 (33.3)
Antimalarials	3 (2.0)	3 (100.0)	0 (0.0)	31.3 (27-40)		0 (0.0)	3 (100.0)	0 (0.0)	0 (0.0)		0 (0.0)	0 (0.0)	0 (0.0)
Hypnotics and sedatives	3 (2.0)	1 (33.3)	2 (66.7)	48.3 (38-59)		0 (0.0)	3 (100.0)	0 (0.0)	3 (100.0)		3 (100.0)	0 (0.0)	1 (33.3)
Anti-Parkinson drugs	2 (1.3)	1 (50.0)	1 (50.0)	48.5 (44-53)		0 (0.0)	2 (100.0)	0 (0.0)	1 (50.0)		1 (50.0)	0 (0.0)	0 (0.0)
Corticosteroids	2 (1.3)	1 (50.0)	1 (50.0)	55.5 (43-68)		0 (0.0)	1 (50.0)	1 (50.0)	1 (50.0)		1 (50.0)	1 (50.0)	0 (0.0)
Cough suppressants	2 (1.3)	1 (50.0)	1 (50.0)	56.0 (46-66)		0 (0.0)	1 (50.0)	1 (50.0)	1 (50.0)		1 (50.0)	0 (0.0)	0 (0.0)
Drugs for obstructive airway diseases	2 (1.3)	1 (50.0)	1 (50.0)	40.0 (9-71)		1 (50.0)	0 (0.0)	1 (50.0)	0 (0.0)		0 (0.0)	0 (0.0)	0 (0.0)
Antimigraine preparations	1 (0.7)	1 (100.0)	0 (0.0)	43.0 (43-43)		0 (0.0)	1 (100.0)	0 (0.0)	1 (100.0)		1 (100.0)	1 (100.0)	0 (0.0)
Antimycotics	1 (0.7)	1 (100.0)	0 (0.0)	67.0 (67-67)		0 (0.0)	0 (0.0)	1 (100.0)	0 (0.0)		0 (0.0)	0 (0.0)	0 (0.0)
Antineoplastic agents	1 (0.7)	0 (0.0)	1 (100.0)	52.0 (52-52)		0 (0.0)	1 (100.0)	0 (0.0)	0 (0.0)		0 (0.0)	0 (0.0)	0 (0.0)
Anxiolytics	1 (0.7)	1 (100.0)	0 (0.0)	62.0 (62-62)		0 (0.0)	0 (0.0)	1 (100.0)	1 (100.0)		1 (100.0)	0 (0.0)	0 (0.0)
Beta-blocking agents	1 (0.7)	1 (100.0)	0 (0.0)	21.0 (21-21)		0 (0.0)	1 (100.0)	0 (0.0)	0 (0.0)		0 (0.0)	0 (0.0)	0 (0.0)
Drugs for constipation	1 (0.7)	0 (0.0)	1 (100.0)	61.0 (61-61)		0 (0.0)	0 (0.0)	1 (100.0)	0 (0.0)		0 (0.0)	0 (0.0)	0 (0.0)
Hormone antagonists and related agents	1 (0.7)	1 (100.0)	0 (0.0)	45.0 (45-45)		0 (0.0)	1 (100.0)	0 (0.0)	1 (100.0)		1 (100.0)	0 (0.0)	1 (100.0)
Propulsive	1 (0.7)	1 (100.0)	0 (0.0)	30.0 (30-30)		0 (0.0)	1 (100.0)	0 (0.0)	0 (0.0)		0 (0.0)	0 (0.0)	0 (0.0)

^a^Number of patients who were duplicated because the cases were associated with more than 2 individual drugs.

^b^The proportion was estimated as the proportion of reported cases within each drug classes.

^c^AI: anti-inflammatory.

^d^AR: antirheumatic.

**Figure 2:**
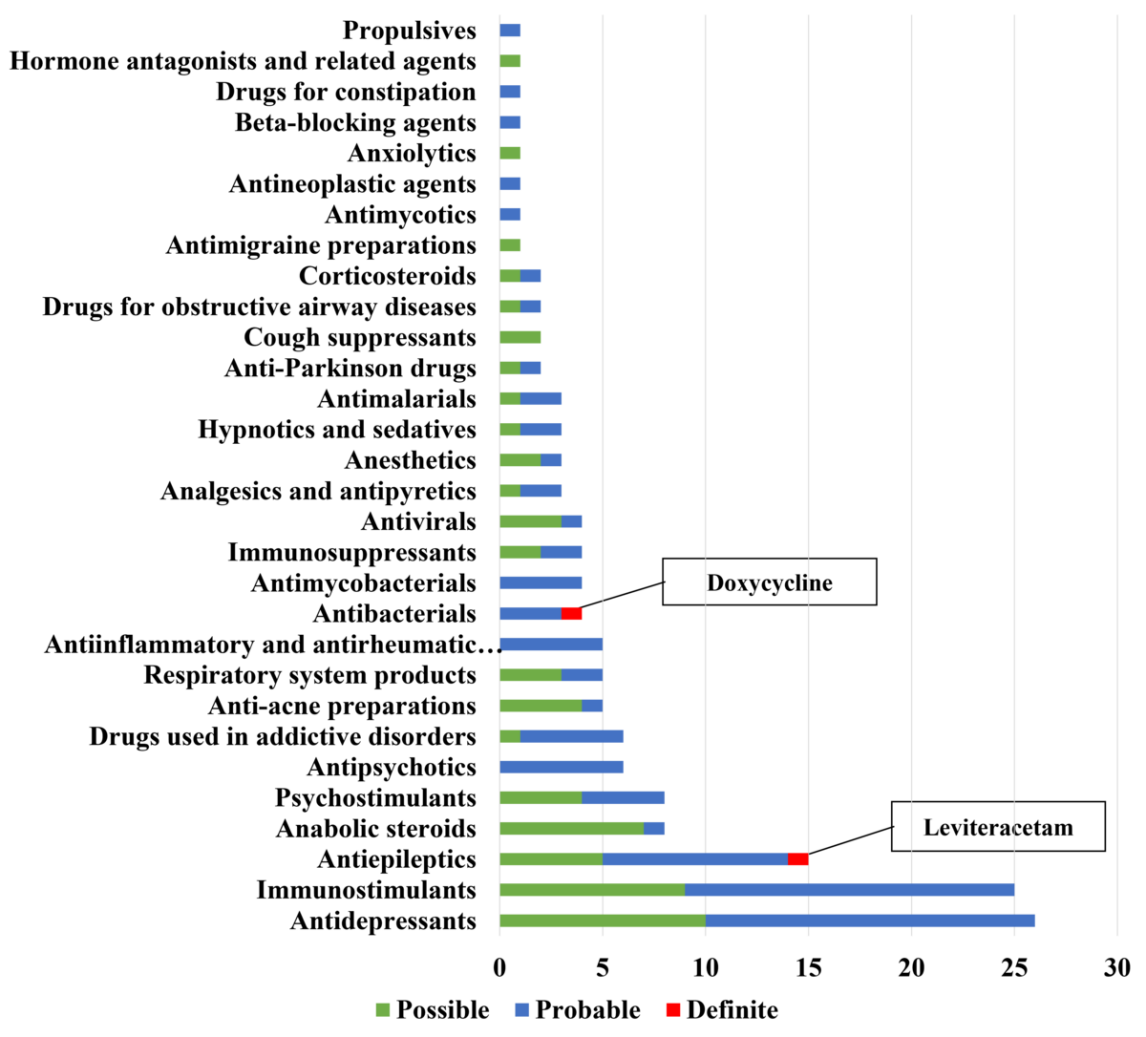
Proportion of the causality assessment for suspected drugs of suicidal adverse drug reactions using the Naranjo algorithm. AI: anti-inflammatory; AR: antirheumatic.

Figure 2 and Table S2 in Multimedia Appendix 2 show the results of the causality assessment of all cases based on the Naranjo algorithm. The results revealed a definite causal association with DIS in 2 cases, probable association in 90 cases, and possible association in 61 cases. The 2 cases with definite causation of DIS were identified in cases associated with doxycycline (antibacterial) and perampanel (antiepileptics), respectively.

Table 2 shows the results of the comparative analysis between our results and the drug labels. The labels of 51 (85%) of 60 drugs contained information regarding the risk of suicidal ADRs. The high- and intermediate-level evidence groups included 33 and 18 drugs, respectively. However, the risk of suicidal ADRs was not indicated on the label of the 9 suspected drugs. Of these, 6 drugs had a “probable” causal relationship (lumacaftor/ivacaftor, doxycycline, piroxicam, clozapine, adalimumab, and paclitaxel), whereas the remaining 3 drugs had “possible” causality (ie, infliximab, dextromethorphan, and formoterol).

**Table 2:** Comparative assessment of suspected drugs in suicidal adverse drug reactions between reviewed case reports and the approved labeling information^a^.

Causality assessment based on the Naranjo algorithm^b^	High (N=33), n	Intermediate (N=18), n	Low (N=9), n
Probable	18	12	6^c^
Possible	15	6	3^d^

^a^In this analysis, we excluded anabolic androgenic steroids because they did not present the information of the involved agent.

^b^Causality was evaluated for each drug based on the average Naranjo scores in the case reports for each drug.

^c^Lumacaftor/ivacaftor, doxycycline, piroxicam, clozapine, adalimumab, and paclitaxel.

^d^Infliximab, dextromethorphan, and formoterol.

The number of cases and the criteria and average score of Naranjo for 17 case reports implicating suspected drugs with low-level evidence on the approved labels were as follows, respectively; lumacaftor/ivacaftor (5 cases, probable 5) doxycycline (3 cases, probable 7.3), piroxicam (1 case, probable 5); clozapine (2 cases, probable 6); adalimumab (2 cases, probable 6.0),; paclitaxel (1 case, probable 5.0), infliximab (1 case, possible 4), dextromethorphan (2 cases, possible 3.5), and formoterol (1 case, possible 3). Multimedia Appendix 5 [21-55] provides detailed information on the evidence of the Naranjo score of each case.

## Discussion

### Principal Findings

To the best of our knowledge, this is the first comprehensive systematic review of DIS based on published case reports. While several studies have reported on the suicide risk of drugs [7-15], most of them have concentrated on limited drugs for which risks have previously been reported. Therefore, our study aimed to bridge this gap by conducting a systematic review of case reports for all available drugs with suicidal risk, including those not previously known to have such a risk. We identified 61 drugs from 29 classes associated with DIS based on published case reports, of which 9 drugs showed a potential risk of suicide despite not being mentioned in their labels.

Most documented DIS cases involved drugs that already had a potential suicidal risk indicated on their labels. This tendency might result from an increased focus on drugs recognized for their potential risk of suicidal ADRs. For instance, antidepressants, the most frequently reported drugs related to suicidal ADRs, carry black box warnings of suicidal risk issued by the FDA. While some drug labels did not directly mention the risk of suicide, they indicated the potential psychiatric ADRs that can lead to suicidal behaviors. Propranolol labels present depression and hallucinations among its psychiatric side effects, which could contribute to triggering suicidal events. Recent studies using pharmacovigilance databases have also strengthened evidence regarding the suicidal risks associated with these drugs [11-15].

This systematic review has found 9 drugs—lumacaftor/ivacaftor, doxycycline, piroxicam, clozapine, paclitaxel, adalimumab, infliximab dextromethorphan, and formoterol—that potentially carry suicidal risks not mentioned on the labels. Despite the absence of available data on labels, previous studies have investigated the potential for suicide or suicide-related behaviors associated with these drugs. However, the precise mechanism related to these suicidal risks remains still unclear. For instance, doxycycline, which exhibited the highest causality for suicidal ADRs in our study, might be related to heightened psychiatric adverse effects due to increased retinoid levels by CYP450 inhibitory effect [21]. In addition, other studies suggested changes in the gut microbiome composition after antibiotic use, leading to the incidence of psychiatric disorders, such as depression and schizophrenia [22-24]. A previous systematic review reported that several clinical trials reported the improvement of depressive, anxiety, and mood symptoms after probiotic treatment [25].

The suicide risk of piroxicam may be related to inhibiting cyclooxygenase activity involved in synthesizing prostaglandins [26,27]. However, there is a lack of direct information about changes in prostaglandin levels [28]. Paclitaxel may be attributed to glutamine depletion and a neurotransmitter [29], even though a clear association with suicide is challenging due to the limited information on patients’ posttreatment status [30]. Other drugs, tumor necrosis factor-α inhibitors [31-35], paclitaxel [29], dextromethorphan [36-38], formoterol [39], clozapine [40-43], and lumacaftor/ivacaftor [44-46] are not easy to explain the clear mechanism for the suicidal events. Despite these complexities, most cases involving these 9 drugs reported symptom improvement after drug discontinuation. Hence, the potential risk of suicide associated with these drugs cannot be disregarded, and further studies are a crucial for clear understanding of the potential suicidal risk of these drugs.

### Limitations

This study also has certain limitations. First, as case reports are not experimental studies conducted with a placebo, the causal relationship between drugs and ADRs cannot be determined. Although we used the Naranjo algorithm to evaluate causality, other factors, such as the underlying psychiatric disorders and other drugs, could affect suicidal events. Therefore, our findings only provide some safety signals regarding which drugs may have a higher risk of suicide not identified in clinical trials [56]. Second, the number of cases for each drug is insufficient for generalizing the findings to the population level. Third, we used PubMed only and did not manually search other journals or sources of gray literature and restricted the inclusion of studies to those published in English. Therefore, our findings might not represent all published case reports. Nevertheless, we constructed a detailed search query using the term of all marketed drugs, which may minimize the impact of limitation. Furthermore, studies have demonstrated that excluding non-English studies might have little effect on the results [57,58].

### Conclusions

Our systematic review of case reports identified a potential risk of suicide associated with several drugs. Most of the cases involved drugs with a documented suicide risk on the label, but 9 drugs were identified without such reporting. Although it is difficult to establish evidence of ADRs based on case reports alone, our findings highlighted the need to raise awareness of the potential for DIS beyond what is documented in drug labels. Therefore, further research exploring suicidal ADRs across various drugs is essential.

## Appendix

Multimedia Appendix 1 PRISMA 2020 checklist.

Multimedia Appendix 2 Supporting methods and results.

Multimedia Appendix 3 Causality assessment using the Naranjo algorithm.

Multimedia Appendix 4 CARE check list.

Multimedia Appendix 5 Summary of cases of suspected drugs without the required information on the approved label.

## Abbreviations

ADR: adverse drug reaction
ATC: anatomical therapeutic chemical
CARE: Case Reports
DIS: drug-induced suicide
FDA: Food and Drug Administration
PRISMA: Preferred Reporting Items for Systematic Reviews and Meta-Analyses
