# Supplementary material for: Assessing the Labeling Information on Drugs Associated With Suicide Risk: Systematic Review
Source: JMIR Public Health Surveill. 2024 Jan 30;10:e49755. doi: 10.2196/49755 (PMC10865198; doi:10.2196/49755)
Supplement: Multimedia Appendix 2 [file publichealth_v10i1e49755_app2.docx]

Drug-induced suicide: a systematic review of case reports and comparison of the safety information with the approved drug label

Soo Min Jeon1†, HyunJoo Lim2†, Hyo-Bin Cheon2, JuHee Ryu2, Jin-Won Kwon3

*** Correspondence:** Jin-Won Kwon: [jwkwon@knu.ac.kr](mailto:jwkwon@knu.ac.kr)

| **Textbox S1.** Detailed search term used to retrieve case reports of DIS in PubMed^a^. |
| --- |
| (%DRUG% [Supplementary concept]OR %DRUG%[mesh Terms]OR %DRUG%[TW]) AND  (“suicidal ideation”[MeSH terms] OR “suicide, attempted”[MeSH terms]  OR “suicide, completed”[MeSH terms] OR “suicide”[MeSH terms]  OR suicide[TW] OR suicidals[TW] OR suicidality[TW] OR suicide[TW]OR suicidal[TW] OR suiciders[TW]OR suicidally[TW] OR suicides[TW]OR suicide s[TW] OR suicided[TW]) AND (case reports[Filter]) AND (English[Language]) |
| Drug-induced suicide, DIS  ^a^For drug keywords, we used the active ingredients and generic names of the drug as keywords for the drugs. The active ingredients or generic names of the drugs were searched on “Drugbank (https://go.drugbank.com/).” According to the Anatomical Therapeutic Chemical Classification System, the following types of medications are not included in this process: vitamins, mineral supplements, tonics, blood substitutes, emollients and protectives, antiseptics and disinfectants, and medicated dressings along with various sections of the classification. |

| **Table S1.** Drug classification according to the ATC code^a^. | |
| --- | --- |
| **Drug class** | **ATC code** |
| Antidepressants | N06A |
| Immunostimulants | L03A |
| Psychostimulants | N06B |
| Antiepileptics | N03A |
| Antipsychotics | N05A |
| Anabolic steroids | A14A |
| Antiinflammatory and antirheumatic products, nonsteroids | M01A |
| Cough suppressants | R05F, R05D |
| Drugs used in addictive disorders | N07B |
| Antiacne preparations ^b^ | D10 (D10A: topical use, D10B: systemic use) |
| Antimalarials | P01B |
| Narcotics |  |
| Respiratory system products | R07A |
| Analgesics and antipyretics | N02B |
| Antibacterials ^c^ | J01 (J01A: tetracyclines, J01M: quinolone antibacterials) |
| Antimycobacterials ^d^ | J04 (No subclassification) |
| Antivirals | J05 |
| Hypnotics and sedatives | N05C |
| Immunosuppressants | L04A |
| Anesthetics ^b^ | N01 (N01A: topical use, N01B: systemic use) |
| Corticosteroids | S03B, S02B, H02B, H02A, D07A, D07B, D07C, and D07X |
| Antimycotics | J02A |
| Anti-Parkinson drugs ^d^ | N04 (No subclassification) |
| Drugs for constipation | A06A |
| Drugs for obstructive airway diseases ^c^ | R03 (R03B, other drugs for obstructive airway diseases and inhalants; R03D, other systemic drugs for obstructive airway diseases) |
| Alimentary tract and metabolism products | A16A |
| Antimigraine preparations | N02C |
| Antineoplastic agents | L01X |
| Anxiolytics | N05B |
| Beta-blocking agents | C07A |
| Hormone antagonists and related agents | L02B |
| Other nervous system drugs | N07X |
| Propulsives | A03F |
| Anatomical Therapeutic Chemical Classification, ATC | |
| ^a^Drugs were classified according to the level 2 of the ATC | |
| ^b^Subcategories are classified according to the purpose of use (local or whole-body purpose) | |
| ^c^The number of patients was small and they could not be categorized into third-level subgroups | |
| ^d^No subcategories after level 2 | |

| **Table S2.** The results of the causality assessment according to the Naranjo ADR score. | | | | | | | | | | | | | |
| --- | --- | --- | --- | --- | --- | --- | --- | --- | --- | --- | --- | --- | --- |
| **Classes of drug** | **Drug** | **N1** | **N2** | **N3** | **N4** | **N5** | **N6** | **N7** | **N8** | **N9** | **N10** | **Naranjo Score** | **Reference ^a^** |
| Anabolic steroids | AAS | Y | Y | N/A | N/A | Y | N/A | N | N/A | N/A | Y | 3 | 1 |
| Anabolic steroids | AAS | Y | Y | N/A | N/A | Y | N/A | N | N/A | N/A | N/A | 2 |  |
| Anabolic steroids | AAS | Y | Y | N/A | N/A | Y | N/A | N | N/A | Y | Y | 4 |  |
| Anabolic steroids | AAS | Y | Y | N/A | N/A | Y | N/A | N | N/A | N/A | Y | 3 |  |
| Anabolic steroids | AAS | Y | Y | N/A | N/A | Y | N/A | N | N/A | N/A | N/A | 2 |  |
| Anabolic steroids | AAS | Y | Y | N/A | Y | Y | N/A | N | N/A | Y | Y | 6 |  |
| Anabolic steroids | AAS | Y | Y | N/A | N/A | Y | N/A | N | N/A | N/A | Y | 3 |  |
| Anabolic steroids | AAS | Y | Y | N/A | N/A | Y | N/A | N | N/A | N/A | Y | 3 |  |
| Analgesics and antipyretics | Ziconotide | Y | Y | N/A | N/A | Y | N/A | N/A | N | N | Y | 3 | 2 |
| Analgesics and antipyretics | Ziconotide | Y | Y | Y | N/A | Y | N/A | N/A | Y | N | Y | 5 |  |
| Analgesics and antipyretics | Nabiximols | Y | Y | Y | N/A | N/A | N/A | N/A | N/A | N/A | Y | 5 | 3 |
| Anesthetics | Ketamine | Y | Y | N/A | N/A | N/A | N/A | Y | N/A | N/A | Y | 5 | 4 |
| Anesthetics | Ketamine | Y | Y | Y | N/A | Y | N/A | N/A | N/A | N/A | Y | 4 | 5 |
| Anesthetics | Ketamine | Y | Y | Y | N/A | Y | N/A | N/A | N/A | N/A | Y | 4 |  |
| Antiacne preparations | Isotretinoin | Y | Y | Y | Y | Y | N/A | N | Y | N/A | N/A | 6 | 6 |
| Antiacne preparations | Isotretinoin | Y | Y | N/A | N/A | Y | N/A | N/A | N/A | N/A | Y | 3 | 7 |
| Antiacne preparations | Isotretinoin | Y | Y | N/A | N/A | Y | N/A | N/A | N/A | N/A | Y | 3 |  |
| Antiacne preparations | Isotretinoin | Y | Y | N/A | N/A | Y | N/A | N/A | N/A | N/A | Y | 3 |  |
| Antiacne preparations | Isotretinoin | Y | Y | N/A | N/A | Y | N/A | N/A | N/A | N/A | Y | 3 | 8 |
| Antibacterials | Ciprofloxacin | Y | Y | Y | Y | N/A | N/A | N | N/A | Y | Y | 8 | 9 |
| Antibacterials | Doxycycline | Y | Y | N/A | N/A | N | N/A | N/A | N/A | N/A | Y | 6 | 10 |
| Antibacterials | Doxycycline | Y | Y | Y | N/A | N | N/A | N/A | N/A | N/A | N/A | 6 |  |
| Antibacterials | Doxycycline | Y | Y | Y | Y | N | N/A | N/A | Y | N/A | Y | 10 |  |
| Antidepressants | Paroxetine | Y | Y | Y | N/A | Y | N/A | N/A | N/A | N/A | N/A | 3 | 11 |
| Antidepressants | Sertraline | Y | Y | Y | N/A | N/A | N/A | N/A | N/A | N/A | N/A | 4 | 12 |
| Antidepressants | Fluoxetine | Y | Y | Y | N/A | Y | N/A | N/A | Y | N | Y | 5 | 13 |
| Antidepressants | Fluoxetine | Y | N | Y | N/A | Y | N/A | N/A | N/A | Y | Y | 2 |  |
| Antidepressants | Fluoxetine | Y | Y | Y | N/A | Y | N/A | N/A | Y | Y | Y | 6 |  |
| Antidepressants | Fluoxetine | Y | Y | Y | N/A | Y | N/A | N/A | Y | N | Y | 5 |  |
| Antidepressants | Fluoxetine | Y | N | Y | N/A | Y | N/A | N/A | Y | N | Y | 2 |  |
| Antidepressants | Fluoxetine | Y | Y | N | N/A | Y | N/A | N/A | N | N | Y | 3 |  |
| Antidepressants | Fluoxetine | Y | Y | N | Y | Y | N/A | N/A | N | N | Y | 5 | 14 |
| Antidepressants | Fluoxetine | Y | Y | N/A | Y | N | N/A | N/A | N/A | N/A | Y | 8 |  |
| Antidepressants | Fluoxetine | Y | Y | N | Y | N/A | N/A | N/A | N | N | Y | 6 |  |
| Antidepressants | Fluoxetine | Y | N | Y | Y | Y | N/A | N/A | N/A | N | Y | 3 |  |
| Antidepressants | Fluoxetine | Y | Y | N/A | N/A | Y | N/A | N/A | N/A | N/A | Y | 3 |  |
| Antidepressants | Fluoxetine | Y | Y | Y | N/A | N/A | N/A | N/A | Y | N/A | Y | 6 |  |
| Antidepressants | Escitalopram | Y | Y | N/A | N/A | N/A | N/A | N/A | N/A | N/A | Y | 4 | 15 |
| Antidepressants | Fluoxetine | Y | Y | Y | N/A | N/A | N/A | N/A | Y | N/A | Y | 6 | 16 |
| Antidepressants | Escitalopram | Y | Y | Y | N/A | N/A | N/A | N/A | N/A | N/A | Y | 5 | 17 |
| Antidepressants | Sertraline | Y | Y | Y | N/A | Y | N/A | N/A | N/A | N/A | Y | 4 | 18 |
| Propulsives | Metoclopramide | Y | Y | Y | N/A | N | N/A | N/A | N/A | N/A | Y | 7 |  |
| Antidepressants | Mirtazapine | Y | Y | Y | N/A | Y | N/A | N/A | Y | N | Y | 5 | 19 |
| Anti-Parkinson drugs | Levodopa | Y | Y | N/A | N/A | Y | N/A | N/A | N/A | N/A | Y | 3 |  |
| Anxiolytics | Benzodiazepines | Y | Y | N | N/A | N/A | N/A | N/A | N | N/A | Y | 4 | 20 |
| Antidepressants | Fluoxetine | Y | Y | Y | N/A | N/A | N/A | N/A | Y | Y | N/A | 6 | 21 |
| Antidepressants | Sertraline | Y | Y | Y | N/A | N | N/A | N | N/A | N/A | Y | 7 | 22 |
| Antidepressants | Bupropion | Y | N/A | N | N/A | Y | N/A | N/A | N | N/A | Y | 1 | 23 |
| Antidepressants | Escitalopram | Y | Y | Y | N/A | Y | N/A | N/A | Y | N/A | Y | 5 | 24 |
| Antiepileptics | Rufinamide | Y | N | Y | N/A | Y | N/A | N/A | Y | N/A | N/A | 1 | 25 |
| Antiepileptics | Rufinamide | Y | N | Y | N/A | Y | N/A | N/A | Y | N/A | N/A | 1 |  |
| Antiepileptics | Perampanel | Y | Y | Y | N/A | N | N/A | N/A | Y | Y | Y | 9 | 26 |
| Antiepileptics | Perampanel | Y | Y | Y | N/A | N | N/A | N/A | Y | N | Y | 8 |  |
| Antiepileptics | Perampanel | Y | Y | Y | N/A | N | N/A | N/A | Y | N | Y | 8 |  |
| Antiepileptics | Ethosuximide | Y | Y | Y | N/A | N | N/A | N/A | Y | N/A | Y | 8 | 27 |
| Antiepileptics | Gabapentin | Y | Y | Y | N/A | N/A | N/A | N/A | Y | N/A | N | 5 | 28 |
| Antiepileptics | Levetiracetam | Y | Y | Y | N/A | N/A | N/A | N/A | N | N | Y | 5 | 29 |
| Antiepileptics | Levetiracetam | Y | Y | N/A | N/A | N/A | N/A | N/A | Y | N/A | Y | 5 | 30 |
| Antiepileptics | Levetiracetam | Y | Y | Y | N/A | Y | N/A | N/A | N/A | N/A | Y | 4 |  |
| Antiepileptics | Levetiracetam | Y | Y | N/A | N/A | N/A | N/A | N/A | Y | N/A | Y | 5 |  |
| Antiepileptics | Levetiracetam | Y | Y | Y | N/A | N | N/A | N/A | N/A | N/A | N/A | 6 |  |
| Antiepileptics | Levetiracetam | Y | Y | Y | N/A | Y | N/A | N/A | N/A | N/A | N/A | 3 |  |
| Antiepileptics | Pregabalin | Y | Y | Y | N/A | N/A | N/A | N/A | N/A | N | Y | 5 | 31 |
| Antiepileptics | Gabapentin | Y | Y | N/A | N/A | Y | N/A | N/A | N/A | N/A | Y | 3 | 32 |
| Antimigraine preparations | Clonidine | Y | Y | N/A | N/A | Y | N/A | N/A | N/A | N/A | Y | 3 |  |
| Corticosteroids | Prednisone | Y | Y | N/A | N/A | Y | N/A | N/A | N/A | N/A | Y | 3 |  |
| AI and AR products, nonsteroids | Piroxicam | Y | Y | Y | N/A | Y | N/A | N/A | N/A | Y | Y | 5 | 33 |
| AI and AR products, nonsteroids | Diclofenac | Y | Y | Y | Y | Y | N/A | N/A | N/A | Y | Y | 7 |  |
| AI and AR products, nonsteroids | Ibuprofen | Y | Y | Y | N/A | Y | N/A | N/A | N/A | Y | Y | 5 |  |
| AI and AR products, nonsteroids | Naproxen | Y | Y | Y | N/A | Y | N/A | N/A | N/A | Y | Y | 5 |  |
| AI and AR products, nonsteroids | Sulindac | Y | Y | Y | Y | Y | N/A | N/A | Y | N | Y | 7 |  |
| Antimalarials | Mefloquine | Y | Y | Y | Y | Y | N/A | N | N/A | Y | Y | 7 | 34 |
| Antimalarials | Chloroquine | Y | Y | N/A | N/A | Y | N/A | Y | N/A | N/A | Y | 4 | 35 |
| Antimalarials | Mefloquine | Y | Y | N/A | N/A | N/A | N/A | Y | N/A | N/A | Y | 5 |  |
| Antimycobacterials | Cycloserine | Y | Y | N/A | N/A | N | N/A | N/A | N/A | N/A | Y | 6 | 36 |
| Antimycobacterials | Isoniazid | Y | Y | Y | N/A | N/A | N/A | N/A | Y | N/A | Y | 6 | 37 |
| Antimycobacterials | Cycloserine | Y | Y | Y | N/A | N/A | N/A | N/A | N/A | N/A | Y | 5 | 38 |
| Antimycobacterials | Cycloserine | Y | Y | Y | N/A | N/A | N/A | N | Y | N/A | Y | 6 | 39 |
| Antimycotics | Voriconazole | Y | Y | Y | N/A | N/A | N/A | Y | N/A | N/A | Y | 6 | 40 |
| Antineoplastic agents | Paclitaxel | Y | Y | Y | N/A | N/A | N/A | N/A | N/A | N | Y | 5 | 41 |
| Anti-Parkinson drugs | Apomorphine | N/A | Y | Y | N/A | Y | N/A | N/A | Y | Y | Y | 5 | 42 |
| Antipsychotics | Aripiprazole | Y | Y | Y | N/A | N/A | N/A | N/A | Y | N | N/A | 5 | 43 |
| Antipsychotics | Aripiprazole | Y | Y | Y | N/A | Y | N/A | N/A | Y | Y | Y | 6 | 44 |
| Antipsychotics | Haloperidol | Y | Y | Y | N/A | N/A | N/A | N/A | N/A | N/A | Y | 5 | 45 |
| Antipsychotics | Ziprasidone | Y | Y | N | N/A | N/A | N/A | N/A | Y | N | Y | 5 | 46 |
| Antipsychotics | Clozapine | Y | Y | Y | N/A | N/A | N/A | N/A | Y | N/A | Y | 6 | 47 |
| Antivirals | Efavirenz | Y | Y | Y | N/A | Y | N/A | N | N/A | N/A | Y | 4 | 48 |
| Antivirals | Oseltamivir | Y | Y | Y | N/A | Y | N/A | N | N/A | N/A | Y | 4 | 49 |
| Antivirals | Ribavirin | Y | Y | N | N/A | Y | N/A | N/A | N/A | N/A | Y | 3 | 50 |
| Immunostimulants | Interferon alpha | Y | Y | N | N/A | Y | N/A | N/A | N/A | N/A | Y | 3 |  |
| Beta-blocking agents | Propranolol | Y | Y | Y | N/A | N | N/A | N/A | Y | N/A | Y | 8 | 51 |
| Corticosteroids | Dexamethasone | Y | Y | Y | N | N/A | N/A | N/A | Y | Y | Y | 6 | 52 |
| Cough suppressants | DXM | Y | Y | N/A | N/A | N/A | N/A | N/A | N/A | N/A | Y | 4 | 53 |
| Cough suppressants | DXM | Y | Y | N/A | N/A | Y | N/A | N | N/A | N/A | Y | 3 | 54 |
| Drugs for constipation | Prucalopride | Y | Y | Y | N/A | N | N/A | N/A | N/A | N/A | N/A | 6 | 55 |
| Drugs for obstructive airway diseases | Formoterol | Y | Y | Y | N/A | Y | N/A | N/A | N/A | N/A | N/A | 3 | 56 |
| Drugs for obstructive airway diseases | Theophylline | Y | Y | Y | N/A | N/A | N/A | Y | Y | N/A | Y | 7 | 57 |
| Drugs used in addictive disorders | Varenicline | Y | Y | Y | Y | Y | N/A | N/A | Y | N/A | N/A | 6 | 58 |
| Drugs used in addictive disorders | Varenicline | Y | Y | Y | N/A | Y | N/A | N/A | Y | N/A | N/A | 4 |  |
| Drugs used in addictive disorders | Varenicline | Y | Y | Y | Y | Y | N/A | N/A | Y | N/A | N/A | 6 |  |
| Drugs used in addictive disorders | Varenicline | Y | Y | Y | N/A | N/A | N/A | N/A | Y | N/A | Y | 6 | 59 |
| Drugs used in addictive disorders | Varenicline | Y | Y | N/A | N/A | N | N/A | N | N/A | N/A | Y | 6 | 60 |
| Drugs used in addictive disorders | Varenicline | Y | Y | Y | N/A | N | N/A | N/A | Y | N | Y | 8 | 61 |
| Hormone antagonists and related agents | Tamoxifen | Y | Y | Y | N/A | Y | N/A | N/A | N/A | N/A | Y | 4 | 62 |
| Hypnotics and sedatives | Suvorexant | Y | Y | Y | N/A | Y | N/A | N/A | Y | N/A | N/A | 4 | 63 |
| Hypnotics and sedatives | Eszopiclone | Y | Y | Y | N/A | Y | N/A | N/A | Y | N | Y | 5 | 64 |
| Hypnotics and sedatives | Zolpidem | Y | Y | Y | N/A | N | N/A | N/A | Y | N/A | Y | 8 | 65 |
| Immunostimulants | Interferon-α | Y | Y | Y | N | Y | N/A | N/A | Y | N/A | Y | 4 | 66 |
| Immunostimulants | Interferon-α | Y | Y | Y | N/A | N | N/A | N/A | N/A | N | Y | 7 | 67 |
| Immunostimulants | Interferon-α | Y | Y | Y | N/A | N | N/A | N/A | N/A | N/A | Y | 7 | 68 |
| Immunostimulants | Interferon-β | Y | Y | Y | N/A | N/A | N/A | N/A | Y | N/A | Y | 6 | 69 |
| Immunostimulants | Interferon-α | Y | Y | Y | N/A | Y | N/A | N/A | N/A | N/A | Y | 4 | 70 |
| Immunostimulants | Interferon-α | Y | Y | N/A | N/A | N/A | N/A | N/A | N/A | N/A | Y | 4 |  |
| Immunostimulants | Interferon-α | Y | Y | Y | N/A | N/A | N/A | N/A | N/A | N/A | Y | 5 |  |
| Immunostimulants | Interferon-α | Y | N/A | Y | N/A | N/A | N/A | N/A | N/A | N | N/A | 2 | 71 |
| Antivirals | Ribavirin | Y | Y | Y | N/A | N/A | N/A | N | N/A | N/A | Y | 5 | 72 |
| Immunostimulants | Interferon-α | Y | Y | N/A | N/A | N/A | N/A | N | N/A | N/A | Y | 4 |  |
| Immunostimulants | Interferon-α | Y | Y | N/A | N/A | N/A | N/A | N/A | N/A | N/A | Y | 4 | 73 |
| Immunostimulants | Interferon-β | Y | Y | Y | N/A | N | N | N/A | N/A | N/A | Y | 8 | 74 |
| Immunostimulants | Interferon-β | Y | Y | Y | N/A | N | N | N/A | N/A | N/A | Y | 8 |  |
| Immunostimulants | Interferon-β | Y | Y | Y | N/A | N | N | N/A | N/A | N/A | Y | 8 |  |
| Immunostimulants | Interferon-β | Y | Y | Y | N/A | N | N | N/A | N/A | N/A | Y | 8 |  |
| Immunostimulants | Interferon-β | Y | Y | Y | N/A | N | N | N/A | N/A | N/A | Y | 8 |  |
| Immunostimulants | Interferon-β | Y | Y | Y | N/A | N | N | N/A | N/A | N/A | Y | 8 |  |
| Immunostimulants | Interferon-β | Y | Y | Y | N/A | N | N | N/A | N/A | N/A | Y | 8 |  |
| Immunostimulants | Interferon-β | Y | Y | Y | N/A | N | N | N/A | N/A | N/A | Y | 8 |  |
| Immunostimulants | Interferon-β | Y | Y | Y | N/A | N | N | N/A | N/A | N/A | Y | 8 |  |
| Immunostimulants | Interferon-β | Y | Y | Y | N/A | N | N | N/A | N/A | N/A | Y | 8 |  |
| Immunostimulants | Interferon-β | Y | Y | Y | N/A | N | N | N/A | N/A | N/A | Y | 8 |  |
| Immunostimulants | Interferon-α | Y | Y | N | N/A | N | N/A | N/A | N/A | N/A | Y | 6 | 75 |
| Immunostimulants | Interferon-α | Y | Y | Y | N/A | Y | N/A | N/A | N/A | N/A | Y | 4 |  |
| Immunostimulants | Interferon-α | Y | Y | N/A | N/A | Y | N/A | N/A | N/A | N/A | Y | 3 |  |
| Immunosuppressants | Natalizumab | Y | Y | Y | N/A | N | N/A | N/A | Y | N | N/A | 7 | 76 |
| Immunosuppressants | Adalimumab | Y | Y | Y | N/A | N | N/A | N/A | Y | N/A | Y | 8 | 77 |
| Immunosuppressants | Adalimumab | Y | Y | N/A | N/A | Y | N/A | N/A | Y | N/A | Y | 4 | 78 |
| Immunosuppressants | Infliximab | Y | Y | Y | N/A | Y | N/A | N/A | N/A | N/A | Y | 4 | 79 |
| Psychostimulants | Atomoxetine | Y | Y | Y | N/A | N/A | N/A | N/A | Y | N/A | N/A | 5 | 80 |
| Psychostimulants | Atomoxetine | Y | Y | N | N/A | N/A | N/A | N/A | Y | N | N/A | 4 | 81 |
| Psychostimulants | Atomoxetine | Y | Y | N | N/A | Y | N/A | N/A | N | N | N/A | 2 |  |
| Psychostimulants | Atomoxetine | Y | Y | Y | N/A | Y | N/A | N/A | Y | N | N/A | 4 |  |
| Psychostimulants | Atomoxetine | Y | Y | Y | N/A | N/A | N/A | N/A | Y | N | N/A | 5 |  |
| Psychostimulants | Atomoxetine | Y | Y | Y | N/A | N/A | N/A | N/A | Y | N | N/A | 5 |  |
| Psychostimulants | Atomoxetine | Y | Y | Y | N/A | N/A | N/A | N/A | Y | N | N/A | 5 |  |
| Psychostimulants | Atomoxetine | Y | Y | Y | N/A | Y | N/A | N/A | Y | N | N/A | 4 |  |
| Respiratory system products | LUM/IVA | Y | Y | Y | N/A | Y | N/A | N/A | N/A | N/A | Y | 4 | 82 |
| Respiratory system products | LUM/IVA | Y | Y | Y | N/A | Y | N/A | N/A | N/A | N/A | Y | 4 |  |
| Respiratory system products | LUM/IVA | Y | Y | Y | N/A | N | N/A | N/A | N/A | N/A | Y | 7 |  |
| Respiratory system products | LUM/IVA | Y | Y | Y | N/A | N | N/A | N/A | N/A | N/A | Y | 7 |  |
| Respiratory system products | LUM/IVA | Y | Y | N | N/A | Y | N/A | N/A | N/A | N/A | Y | 3 |  |
| Antidepressants | Fluoxetine | Y | Y | Y | Y | Y | N/A | N/A | Y | Y | Y | 8 | 83 |
| Antidepressants | Fluoxetine | Y | Y | Y | Y | Y | N/A | N/A | Y | Y | Y | 8 |  |
| Antidepressants | Fluoxetine | Y | Y | Y | Y | Y | N/A | N/A | Y | Y | Y | 8 |  |
| Adverse drug reaction, ADR; anabolic androgenic steroids, AAS; antiinflammatory, AI; antirheumatic, AR; dextromethorphan, DXM; lumacaftor/ivacaftor, LUM/IVA; Yes, Y; No, N; Do not know, N/A  a. A list of reference is presented at the end of supplemental material. | | | | | | | | | | | | | |
