# Supplementary material for: Assessing the Labeling Information on Drugs Associated With Suicide Risk: Systematic Review
Source: JMIR Public Health Surveill. 2024 Jan 30;10:e49755. doi: 10.2196/49755 (PMC10865198; doi:10.2196/49755)
Supplement: Multimedia Appendix 3 [file publichealth_v10i1e49755_app3.docx]

**Causality assessment using the Naranjo algorithm**

We used the Naranjo algorithm (Table 1) to assess the causal relationship between medication use and the development of suicide-related symptoms (Naranjo et al., 1981). As some of the questions, such as questions 1, 3, and 10 in the Naranjo algorithm, were not detailed enough to adequately assess causality, we defined detailed criteria (Table 2) and used these criteria to evaluate the Naranjo algorithm.

| **Table S1.** Causality assessment using the Naranjo algorithm. | | | |
| --- | --- | --- | --- |
| Question | Yes | No | Do not know |
| 1. Are there previous conclusion reports on this reaction? | 1 | 0 | 0 |
| 2. Did the adverse reaction occur after the suspected drug was administered? | 2 | −1 | 0 |
| 3. Did the adverse reaction improve when the drug was discontinued or a specific antagonist was administered? | 1 | 0 | 0 |
| 4. Did the adverse reaction reappear when the drug was readministered? | 2 | −1 | 0 |
| 5. Are there alternate causes that could have solely resulted in the reaction? | −1 | 2 | 0 |
| 6. Did the reaction reappear when a placebo was given? | −1 | 1 | 0 |
| 7. Was the drug detected in the blood in a concentration known to be toxic? | 1 | 0 | 0 |
| 8. Was the reaction more severe when the dose was increased or less severe when the dose was decreased? | 1 | 0 | 0 |
| 9. Did the patient have a similar reaction to the same or similar drugs in any previous exposure? | 1 | 0 | 0 |
| 10. Was the adverse event confirmed by objective evidence? | 1 | 0 | 0 |

| **Table S2.** Interpretation of selected questions in the Naranjo algorithm based on detailed criteria^a^. | | |
| --- | --- | --- |
|  | Original Naranjo algorithm | Detailed criteria |
| Q1 | Are there previous conclusion reports on this reaction? | If the adverse effects were listed on the drug label or cases of experiencing these effects were reported in the literature, it was evaluated as Yes (+1). |
| Q3 | Did the adverse reaction improve when the drug was discontinued or a specific antagonist was administered? | If the adverse effects improved after discontinuing the administration of suspected drugs despite recurrence or deterioration of mental illness, it was recognized as recovery immediately after discontinuation; therefore, we evaluated it as Yes (+1). |
| Q10 | Was the adverse event confirmed by objective evidence? | 1) When validated assessments such as the HAM-D depression evaluation scale were performed or 2) diagnosis or hospitalization due to suicide-related adverse effects or 3) suicide was performed, we evaluated it as Yes (+1). |
| Question, Q; Hamilton depression rating scale, HAM-D  A. For criteria that are not presented in this table, we used the existing version of the Naranjo algorithm. | | |
