# Supplementary material for: Assessing the Labeling Information on Drugs Associated With Suicide Risk: Systematic Review
Source: JMIR Public Health Surveill. 2024 Jan 30;10:e49755. doi: 10.2196/49755 (PMC10865198; doi:10.2196/49755)
Supplement: Multimedia Appendix 5 [file publichealth_v10i1e49755_app5.docx]

**Summary of cases of suspected drugs without the required information on the approved label**

*Lumacaftor/ivacaftor*

We identified five cases (reported in one case series) associated with lumacaftor/ivacaftor in adolescent women with cystic fibrosis [50] (Table 1). The causality score for these cases ranged from 3 to 8, the mean value of which was classified as “probable” (5.0). Of these, three patients had a history of psychiatric diagnoses such as anxiety and depression; however, none of them had a history of suicide attempts. Within 2 weeks to 9 months of initiating the administration of lumacaftor/ivacaftor, two patients experienced suicidal ideation and three patients attempted suicide. Moreover, all of them demonstrated concomitant worsening of psychiatric symptoms, including increased anxiety and symptoms of depression. After discontinuing the treatment, four patients showed an improvement in their emotional statuses. However, in one patient with underlying psychiatric comorbidity, there was no improvement in the symptoms of depression and anxiety.

Lumacaftor/ivacaftor is a drug combination that targets the cystic fibrosis transmembrane regulator (CFTR) that is approved for patients with cystic fibrosis. Although CFTR is mainly expressed in specialized epithelial cells, it has also been found in various tissues, including the brain [44]. As a result, the effect of lumacaftor/ivacaftor on CFTR in the central nervous system (CNS) might cause psychiatric disorders. A recent systematic review reported that several cohort, survey, and case series studies demonstrated an increased risk of mental health issues associated with all CFTR modulator therapies, although no findings were reported for lumacaftor/ivacaftor [45]. Furthermore, lumacaftor induces cytochrome P450 (CYP) 3A, 2C9, and 2C19, which are major metabolites of selective serotonin reuptake inhibitors [46]. This suggests that the concomitant use of lumacaftor and antidepressants reduces the efficacy of antidepressants, potentially triggering suicidal ideation. Notably, one case associated with antidepressant use has been identified.

**Table 1. Causality assessment for lumacaftor/ivacaftor based on the Naranjo algorithm.**

| **No** | **Age, sex** | **Dosage** | **Description of event** | **Improvement after discontinuation** | **Recurrence after re-administration** | **Presence of other risk factors for suicide** | **Recurrence after placebo administration** | **Drug levels in the blood /body fluids** | **Change in ADR with dosage** | **History of ADR to identical or similar drug types** | **Objectivity of evidence** | **Naranjo score** |
| --- | --- | --- | --- | --- | --- | --- | --- | --- | --- | --- | --- | --- |
| 1 | 15, F |  | Aggravated: increased anxiety, symptoms of depression, and suicidal ideation (2 weeks) | Yes (within 10 days, sertraline 25 mg ) | N/A | Yes  (anxiety) | N/A | N/A | N/A | N/A | Yes | 4 |
| 2 | 17, F |  | Aggravated: worsening depression and suicidal ideation (2 months) | Yes (fluoxetine 40 mg + CBT) | N/A | Yes  (anxiety and depression) | N/A | N/A | N/A | N/A | Yes | 4 |
| 3 | 14, F |  | Severely aggravated: symptoms of depression (after 9 months) and suicide attempt (after 11 months) | Yes (fluoxetine 20 mg + CBT) | N/A | No | N/A | N/A | N/A | N/A | Yes | 7 |
| 4 | 12, F |  | Severely aggravated: symptoms of depression and suicide attempt (2 months) | Yes (within 3 weeks, sertraline 12.5 mg + CBT) | N/A | No | N/A | N/A | N/A | N/A | Yes | 7 |
| 5 | 17, F |  | Aggravated: worsening depression and suicide attempt (7 months) | No (escitalopram 10 mg + mirtazapine 15 mg + fluoxetine 20 mg) | N/A | Yes (anxiety and depression) | N/A | N/A | N/A | N/A | Yes | 3 |
| **Average score for Naranjo causality** | | | | | | | | | | | | **5.0**  **(Probable)** |
| Male, M; female, F | | | | | | | | | | | | |

*Doxycycline*

We identified three different DIS cases involving doxycycline, which was initially administered for perioral dermatitis or acne [51]. Among them, two patients died from completed suicide and one patient experienced suicidal ideations. The mean Naranjo score for this drug was 7.3 (classified as “probable”), and one case was classified as “definite” causation with the highest score of 10. In this case, the patient had no personal or family history of psychiatric disorders, substance abuse, or suicide attempts; however, he experienced depressive symptoms after the administration of doxycycline (50 mg/day) for acne treatment for >1 year. After discontinuing the treatment, his depressive symptoms improved; however, he died from completed suicide 8 weeks after readministering doxycycline.

While the association between suicide and doxycycline remains inconclusive, doxycycline induced suicidal ADRs might be explained by the increase in retinoid levels caused by doxycycline. Retinoids are compounds associated with an increased risk of depression and suicidal ideation, and doxycycline acts as an inhibitor of CYP450s, the enzyme that metabolizes retinoids [21]. In our review, we identified one case report by Atigari et al. (2013) that described that a patient had CYP450 polymorphisms with decreased activity, particularly of CYP2C19. Meanwhile, other studies have suggested an association between changes in the gut microbiome composition after antibiotic use, leading to the incidence of psychiatric disorders, such as depression and schizophrenia [22-24]. A previous systematic review reported that several clinical trials reported the improvement of depressive, anxiety, and mood symptoms after probiotic treatment [25]

**Table 2. Causality assessment for doxycycline based on the Naranjo algorithm.**

| **No** | **Age, sex** | **Dosage** | **Description of event** | **Improvement after discontinuation** | **Recurrence after re-administration** | **Presence of other risk factors for suicide** | **Recurrence after placebo administration** | **Drug levels in the blood /body fluids** | **Change in ADR with dosage** | **History of ADR to identical or similar drug types** | **Objectivity of evidence** | **Naranjo score** |
| --- | --- | --- | --- | --- | --- | --- | --- | --- | --- | --- | --- | --- |
| 1 | 19,M | 50 mg twice daily | After 6 days | N/A | N/A | Yes (patient’s brother experienced anxiety symptoms after administering doxycycline) | N/A | N/A | N/A | N/A | Yes  (CYP2C19*2 heterozygote genotype) | 6 |
| 2 | 33,F | Increased dosage from 200 mg/day to 100 mg once daily | - Psychiatric adverse effects: after 2 days - Suicidal ideation: after 3 days | Yes  (return to normal without psychotropic drugs) | N/A | No | N/A | N/A | N/A | N/A | N/A | 6 |
| 3 | 18,M | 100 mg/day; he had a medical history of administration of doxycycline [50 mg/day] for >1 year | - Mood down: after taking doxycycline for >1 year - Suicide: after 8 weeks of readministration | Yes | Yes | No | N/A | N/A | Yes  (the occurrence of suicide was noted after restarting treatment at a dosage of 100 mg/day) | Yes | Yes | 10 |
| **Average score for Naranjo causality** | | | | | | | | | | | | **7.3**  **(Probable)** |
| Male, M; female, F | | | | | | | | | | | | |

*Piroxicam*

One case associated with piroxicam was reported to have a probable (5.0) causal relationship with suicidal adverse drug reactions (ADRs) [28] (Table 3). This case was reported in a 37-year-old man with a history of depression for at least 10 years. The patient had a medical history of psychiatric symptoms associated with diclofenac, which is a nonsteroidal anti-inflammatory drug. He received piroxicam (20 mg/day) for treating rheumatoid arthritis and developed suicidal ideation symptoms with agitation, depression, and insomnia. These symptoms were completely resolved within 7 days after discontinuing the drug.

Piroxicam exerts its anti-inflammatory effects by inhibiting cyclooxygenase (COX) activity, involving in synthesizing prostaglandins. Previous studies have suggested a potential association between prostaglandins and psychotic symptoms following the administration of nonsteroidal anti-inflammatory drugs, particularly type 2 prostaglandins produced by COX-2. [26] Therefore, piroxicam-induced suicidal ADRs might be related to its inhibition of COX-2 activity. [27] However, it is important to note that the absence of available data regarding prostaglandin levels in the mentioned case report. [28]

**Table 3. Causality assessment for piroxicam based on the Naranjo algorithm.**

| **No** | **Age, sex** | **Dosage** | **Description of event** | **Improvement after discontinuation** | **Recurrence after re-administration** | **Presence of other risk factors for suicide** | **Recurrence after placebo administration** | **Drug levels in the blood /body fluids** | **Change in ADR with dosage** | **History of ADR to identical or similar drug types** | **Objectivity of evidence** | **Naranjo score** |
| --- | --- | --- | --- | --- | --- | --- | --- | --- | --- | --- | --- | --- |
| 1 | 37, M | 20 mg/day) | Exact onset time was not reported | Yes (after 1 month, fluoxetine 20 mg/day) | Yes | Yes  (major depression for >10 years) | N/A | N/A | N/A | Yes | Yes | 5 |
| **Average score for Naranjo causality** | | | | | | | | | | | | **5**  **(Probable)** |
| Male, M; female, F | | | | | | | | | | | | |

*Clozapine*

We identified one case associated with clozapine, which was observed after increasing the dosage of the drug [52] (Table 4). A 54-year-old man with bipolar disorder (treated for >15 years) experienced suicidal ideations within a week after increasing the dosage of clozapine from 150 mg/day to 300 mg/day (he developed depression, anxiety, and insomnia concomitantly). The patient had multiple records of hospitalizations due to psychiatric disorders and alcohol abuse. This case was considered as having a probable (6.0) causal relationship with suicidal ADRs.

Clozapine is a drug representatively used to reduce the risk of suicide in patients with schizophrenia. However, this drug also has a potential risk of obsessive–compulsive symptoms (OCS), which are a risk factor for suicidal ADRs. [40] A case report included in our study suggested that suicidal obsessions experienced by the patient are a symptom associated with OCS. [41] Another study conducted by Szmulewicz et al. (2015) has found that eight of nine patients who attempted suicide while being treated with clozapine had experienced OCS. [42] Clozapine might change the serotonergic neurotransmission caused by the antagonistic properties of 5-HT1C, 5-HT2A, and 5-HT2C receptors, which can lead to psychiatric ADRs. [43] Therefore, monitoring OCS after administering clozapine might be critical for reducing suicidal ADRs.

**Table 4. Causality assessment for clozapine based on the Naranjo algorithm.**

| **No** | **Age, sex** | **Dosage** | **Description of event** | **Improvement after discontinuation** | **Recurrence after re-administration** | **Presence of other risk factors for suicide** | **Recurrence after placebo administration** | **Drug levels in the blood /body fluids** | **Change in ADR with dosage** | **History of ADR to identical or similar drug types** | **Objectivity of evidence** | **Naranjo score** |
| --- | --- | --- | --- | --- | --- | --- | --- | --- | --- | --- | --- | --- |
| 1 | 54, M | From 150 mg/day to 300 mg/day | Suicidal obsessions (in the week after increasing dose of clozapine) | Yes (after few days, changing a treatment as quetiapine) | N/A | N/A | N/A | N/A | Yes (the suicidal symptoms were developed within few weeks after increasing the dosage of clozapine to 300 mg/day) | N/A | Yes | 6 |
| **Average score for Naranjo causality** | | | | | | | | | | | | **6**  **(Probable)** |
| Male, M; female, F | | | | | | | | | | | | |

*Adalimumab and infliximab*

Table 5 and Table 6 present the causality assessment of cases regarding adalimumab and infliximab. Two cases associated with adalimumab were observed after increasing the dosage of the drug. The Naranjo causality was classified as “probable” [35] and “possible,” [53] respectively. The dosage of adalimumab was increased in a 56-year-old woman from 40 mg/2 weeks to 40 mg/week for treating rheumatoid arthritis [35]. Although the patient had no personal or family history of increased suicidal risk, she experienced suicidal ideation during this treatment. Furthermore, she experienced symptoms of anti-TNF-α–induced lupus (ATIL), such as facial rash, shoulder pain, and oral ulcers. The other case associated with the administration of adalimumab was reported in a 53-year-old man with chronic psoriasis for >30 years [53]. After 1 year of adalimumab treatment at a dosage of 40 mg/week, his symptoms of psoriasis relapsed. Considering that he experienced no side effects during the initial adalimumab treatment and was obese, he received twice the original dose of adalimumab. After reloading the dose, his symptoms of psoriasis were well controlled without any side effects; however, he suddenly died from suicide 2 months later. Before initiating adalimumab treatment, he already had a low mood disorder and attempted suicide several times. A case associated with infliximab was observed in a 43-year-old man with ulcerative colitis [54]. He developed depression and attempted suicide after receiving infliximab. There were no personal or familial risk factors for suicide. The causality assessment for these cases was performed as soon as possible.

A systematic review and meta-analysis of RCTs and cohort studies found no evidence of increased risk of neuropsychiatric adverse effects associated with tumor necrosis factor-alpha (TNF-α) inhibitors. [31] However, there were three cases of suicidal ADRs associated with the administration of TNF-α inhibitors, including adalimumab and infliximab. Figiel et al. (2008) have reported that TNF-α exerts neuroprotective effects on cerebral neuropathology. [32] Therefore, the reduction in TNF-α levels induced by these drugs might affect neuropsychiatric systems, leading to the development of suicidal ADRs. However, this potential mechanism has only been limited to animal studies. [33,34] Furthermore, one case report [35] has suggested that the occurrence of suicidal attempts is linked to angioimmunoblastic T cell lymphoma (ATIL) induced by adalimumab. However, considering the low incidence of ATIL in patients treated with adalimumab (0.10%) and the rarity of neuropsychiatric symptoms associated with ATIL [35], it is unlikely to explain the association between TNF-α inhibitors and suicidal ADRs.

**Table 5. Causality assessment for adalimumab based on the Naranjo algorithm.**

| **No** | **Age, sex** | **Dosage** | **Description of event** | **Improvement after discontinuation** | **Recurrence after re-administration** | **Presence of other risk factors for suicide** | **Recurrence after placebo administration** | **Drug levels in the blood /body fluids** | **Change in ADR with dosage** | **History of ADR to identical or similar drug types** | **Objectivity of evidence** | **Naranjo score** |
| --- | --- | --- | --- | --- | --- | --- | --- | --- | --- | --- | --- | --- |
| 1 | 56, F | from 40 mg SC/2 weeks to 40 mg SC/week | Suicidal ideations, rash, and joint pain (2 weeks after increasing adalimumab dosage) | Yes (after a few weeks, treating with a course of glucocorticoids) | N/A | No | N/A | N/A | Yes | N/A | N/A | 8 |
| 2 | 53, M | from 80 mg loading dose to 40 mg/2 weeks; the dosage was then doubled due to the relapse of psoriasis | No side effects were reported because of the administration of adalimumab before he committed suicide after 2 months: suicide | N/A | N/A | Yes (history of mental illness including suicidal ideation) | N/A | N/A | Yes | N/A | Yes | 4 |
| **Average score for Naranjo causality** | | | | | | | | | | | | **6**  **(Probable)** |
| Male, M; female, F | | | | | | | | | | | | |

**Table 6. Causality assessment for infliximab based on the Naranjo algorithm.**

| **No** | **Age, sex** | **Dosage** | **Description of event** | **Improvement after discontinuation** | **Recurrence after re-administration** | **Presence of other risk factors for suicide** | **Recurrence after placebo administration** | **Drug levels in the blood /body fluids** | **Change in ADR with dosage** | **History of ADR to identical or similar drug types** | **Objectivity of evidence** | **Naranjo score** |
| --- | --- | --- | --- | --- | --- | --- | --- | --- | --- | --- | --- | --- |
| 1 | 43, M | 4 infusions/day | 4 weeks after his last infusion: negative thoughts increased.  5 weeks after his last infusion: a suicide attempt | Yes | N/A | Yes (work-related stress) | N/A | N/A | N/A | N/A | Yes | 4 |
| **Average score for Naranjo causality** | | | | | | | | | | | | **4**  **(Possible)** |
| Male, M; female, F | | | | | | | | | | | | |

*Paclitaxel*

One case associated with paclitaxel was reported in a 52-year-old woman who received chemotherapy with paclitaxel plus the granulocyte colony-stimulating factor for metastatic breast cancer [29] (Table 7). For this patient, six cycles of chemotherapy were planned, and suicidal depression was noted on day 10 of the last two cycles of paclitaxel. Clinicians discontinued this chemotherapy and newly initiated hormonal therapy. Suicidal depression was improved after the cessation of chemotherapy with paclitaxel. The causality assessment result was “probable.”

The improvement of suicidal depression symptoms after discontinuing paclitaxel treatment suggested a potential association between paclitaxel and suicidal ADRs. In addition, paclitaxel has also been reported to be associated with changes in neurobiochemical metabolites that might be related to the severity of suicidal symptoms. [29,55] However, it is difficult for this hypothesis to explain clear association between paclitaxel and suicidal ADRs because changes in neurochemical metabolites are observed in stressful conditions themselves, including cancer. [30] In mentioned case report, there is no available data regarding the post-treatment status concerning cancer.

**Table 7. Causality assessment for paclitaxel based on the Naranjo algorithm.**

| **No** | **Age, sex** | **Dosage** | **Description of event** | **Improvement after discontinuation** | **Recurrence after re-administration** | **Presence of other risk factors for suicide** | **Recurrence after placebo administration** | **Drug levels in the blood /body fluids** | **Change in ADR with dosage** | **History of ADR to identical or similar drug types** | **Objectivity of evidence** | **Naranjo score** |
| --- | --- | --- | --- | --- | --- | --- | --- | --- | --- | --- | --- | --- |
| 1 | 52, F | 6 cycles of chemotherapy, 1 cycle, 21 days: taxol 125 mg/m^2^ IV over 3 h every 21 days + cimetidine, benadryl, decardron + neupogen 300 µg SC/day, from day 2 to day 10,11, or 12 | Depression and severe agitation with suicide ideation (after 10 days) | Yes (she did not experience depression while receiving hormonal therapy) | N/A | N/A | N/A | N/A | N/A | No | Yes | **5** |
| **Average score for Naranjo causality** | | | | | | | | | | | | **5**  **(Probable)** |
| Male, M; female, F | | | | | | | | | | | | |

*Dextromethorphan*

Two cases associated with dextromethorphan were identified and classified as “possible.” The first case was observed in a 47-year-old woman who experienced suicide attempts [56] (Table 8). The patient took dextromethorphan to control her withdrawal symptoms from oxycodone abuse. According to the patient’s statement, she found the information on the internet that dextromethorphan cough syrup is effective in improving opiate withdrawal symptoms. After the discontinuation of drug administration, her symptoms improved. She had a history of mood disorders and substance abuse (methamphetamine and oxycodone). The other case was associated with an overdose of dextromethorphan [57]. A 66-year-old man ingested cough syrup containing dextromethorphan using a tablespoon (1575 mg/day dextromethorphan) instead of a teaspoon. The patient received sertraline (50 mg/day) for 2 days, and his symptoms completely improved after several months. He had no personal or family history of psychiatric disorders, substance abuse, or suicide attempts.

Dextromethorphan can induce neuropsychiatric adverse events due to its activity as an N-methyl-D-aspartate receptor antagonist [36] and 5-HT1 agonist. [37] Although no information on the risk of suicide was included in the side effects section, this risk is indicated in the overdose section. [38] In two observed cases of suicide attempts, patients had either abused or overdosed on dextromethorphan. Given that dextromethorphan is a common over-the-counter drug in most countries, it is important to note the potential risk of suicide related to this drug.

**Table 8. Causality assessment for dextromethorphan based on the Naranjo algorithm.**

| **No** | **Age, sex** | **Dosage** | **Description of event** | **Improvement after discontinuation** | **Recurrence after re-administration** | **Presence of other risk factors for suicide** | **Recurrence after placebo administration** | **Drug levels in the blood /body fluids** | **Change in ADR with dosage** | **History of ADR to identical or similar drug types** | **Objectivity of evidence** | **Naranjo score** |
| --- | --- | --- | --- | --- | --- | --- | --- | --- | --- | --- | --- | --- |
| 1 | 46, F | Three to four 8-ounce bottles of cough syrup with DXM 10mg | N/A | N/A | N/A | Yes(oxycodone addiction) | N/A | N/A | N/A | N/A | Yes | 3 |
| 2 | 66, M | 1575 mg/day | N/A | N/A | N/A | N/A | N/A | No | N/A | N/A | Yes | 4 |
| **Average score for Naranjo causality** | | | | | | | | | | | | **3.5**  **(Possible)** |
| Male, M; female, F | | | | | | | | | | | | |

*Formoterol*

This case was observed in a 9-year-old boy who was treated for asthma using budesonide plus formoterol [58] (Table 9). Within 28 days of switching the treatment from budesonide (180 mg/puff) two puffs twice daily to budesonide (160 mg/puff) plus formoterol (4.5 mg/puff) two puffs twice daily, the patient experienced mood disorders and subsequent suicidal ideation. These symptoms disappeared within 2 days after switching the treatment back to budesonide monotherapy. There was no personal or family history of psychiatric disorders, substance abuse, or suicide attempts. This case was classified as “possible” causation of DIS. Formoterol-induced suicidal ADRs might be influenced by the stimulation of β2-receptors in the CNS, which is involved in the development of depression, due to the structural similarity of formoterol to catecholamines. [39]

**Table 9. Causality assessment for formoterol based on the Naranjo algorithm.**

| **No** | **Age, sex** | **Dosage** | **Description of event** | **Improvement after discontinuation** | **Recurrence after re-administration** | **Presence of other risk factors for suicide** | **Recurrence after placebo administration** | **Drug levels in the blood /body fluids** | **Change in ADR with dosage** | **History of ADR to identical or similar drug types** | **Objectivity of evidence** | **Naranjo score** |
| --- | --- | --- | --- | --- | --- | --- | --- | --- | --- | --- | --- | --- |
| 1 | 9, M | N/A | <14 days: mood change (yelling, screaming, crying, and episodes of anger)  <28 days: insomnia began approximately 10 days before the onset of suicidal thoughts (20 days after the medication was changed to budesonide + formoterol) | Yes (within 48 h after changing medication from 2^nd^ to 3^rd^: no further mentions of suicide.  after >6 months: he remained free of suicidal ideation) | N/A | Yes (asthma) | N/A | N/A | N/A | N/A | N/A | 3 |
| **Average score for Naranjo causality** | | | | | | | | | | | | **3**  **(Possible)** |
| Male, M; female, F | | | | | | | | | | | | |

**Reference**

1. McKinzie CJ, Goralski JL, Noah TL, Retsch-Bogart GZ, Prieur MB. Worsening anxiety and depression after initiation of lumacaftor/ivacaftor combination therapy in adolescent females with cystic fibrosis. J Cyst Fibros. 2017 Jul;16(4):525-7. PMID: 28602538. doi: 10.1016/j.jcf.2017.05.008.

2. Schneider EK, McQuade RM, Carbone VC, Reyes-Ortega F, Wilson JW, Button B, et al. The potentially beneficial central nervous system activity profile of ivacaftor and its metabolites. ERJ Open Res. 2018 Jan;4(1). PMID: 29560360. doi: 10.1183/23120541.00127-2017.

3. Arslan M, Chalmers S, Rentfrow K, Olson JM, Dean V, Wylam ME, et al. Suicide attempts in adolescents with cystic fibrosis on Elexacaftor/Tezacaftor/Ivacaftor therapy. J Cyst Fibros. 2023 Feb 7. PMID: 36759252. doi: 10.1016/j.jcf.2023.01.015.

4. Schneider EK. Cytochrome P450 3A4 Induction: Lumacaftor versus Ivacaftor Potentially Resulting in Significantly Reduced Plasma Concentration of Ivacaftor. Drug metabolism letters. 2018;12(1):71-4. PMID: 29595119. doi: 10.2174/1872312812666180328105259.

5. Atigari OV, Hogan C, Healy D. Doxycycline and suicidality. BMJ Case Rep. 2013 Dec 17;2013. PMID: 24347450. doi: 10.1136/bcr-2013-200723.

6. Regen F, Hildebrand M, Le Bret N, Herzog I, Heuser I, Hellmann-Regen J. Inhibition of retinoic acid catabolism by minocycline: evidence for a novel mode of action? Experimental dermatology. 2015 Jun;24(6):473-6. PMID: 25810318. doi: 10.1111/exd.12692.

7. Sharon G, Sampson TR, Geschwind DH, Mazmanian SK. The Central Nervous System and the Gut Microbiome. Cell. 2016 Nov 3;167(4):915-32. PMID: 27814521. doi: 10.1016/j.cell.2016.10.027.

8. Kelly JR, Borre Y, C OB, Patterson E, El Aidy S, Deane J, et al. Transferring the blues: Depression-associated gut microbiota induces neurobehavioural changes in the rat. Journal of psychiatric research. 2016 Nov;82:109-18. PMID: 27491067. doi: 10.1016/j.jpsychires.2016.07.019.

9. Bastiaanssen TFS, Cowan CSM, Claesson MJ, Dinan TG, Cryan JF. Making Sense of ... the Microbiome in Psychiatry. Int J Neuropsychopharmacol. 2019 Jan 1;22(1):37-52. PMID: 30099552. doi: 10.1093/ijnp/pyy067.

10. Wallace CJK, Milev R. The effects of probiotics on depressive symptoms in humans: a systematic review. Ann Gen Psychiatr. 2017 Feb 20;16. PMID: WOS:000394466600001. doi: ARTN 14

10.1186/s12991-017-0138-2.

11. Jiang HK, Chang DM. Non-steroidal anti-inflammatory drugs with adverse psychiatric reactions: five case reports. Clin Rheumatol. 1999;18(4):339-45. PMID: 10468178. doi: 10.1007/s100670050114.

12. Famitafreshi H, Karimian M. Prostaglandins as the Agents That Modulate the Course of Brain Disorders. Degener Neurol Neuromuscul Dis. 2020;10:1-13. PMID: 32021549. doi: 10.2147/DNND.S240800.

13. Onder G, Pellicciotti F, Gambassi G, Bernabei R. NSAID-related psychiatric adverse events: who is at risk? Drugs. 2004;64(23):2619-27. PMID: 15537366. doi: 10.2165/00003495-200464230-00001.

14. Aukst-Margetić B, Margetić B, Marsanić VB. Suicidal obsessions as dose dependent side-effect of clozapine. Psychopharmacology bulletin. 2011;44(1):65-9. PMID: 22506440.

15. Grover S, Hazari N, Chakrabarti S, Avasthi A. Relationship of obsessive compulsive symptoms/disorder with clozapine: A retrospective study from a multispeciality tertiary care centre. Asian J Psychiatr. 2015 Jun;15:56-61. PMID: 26013671. doi: 10.1016/j.ajp.2015.05.002.

16. Aukst-Margetić B, Margetić B, Maršanić VB. Suicidal obsessions as dose dependent side-effect of clozapine. Psychopharmacology Bulletin. 2011;44(1):65.

17. Szmulewicz AG, Smith JM, Valerio MP. Suicidality in clozapine-treated patients with schizophrenia: role of obsessive-compulsive symptoms. Psychiatry research. 2015;230(1):50-5.

18. Schirmbeck F, Zink M. Clozapine-induced obsessive-compulsive symptoms in schizophrenia: a critical review. Current Neuropharmacology. 2012;10(1):88-95.

19. Jafri F, Sammut A. A rare case of suicidal ideation related to Adalimumab use. Open Access Rheumatol. 2018;10:113-5. PMID: 30147384. doi: 10.2147/oarrr.S168559.

20. Ellard R, Ahmed A, Shah R, Bewley A. Suicide and depression in a patient with psoriasis receiving adalimumab: the role of the dermatologist. Clinical and Experimental Dermatology. 2014 2014/07/01;39(5):624-7. doi: https://doi.org/10.1111/ced.12351.

21. Eshuis EJ, Magnin KM, Stokkers PC, Bemelman WA, Bartelsman J. Suicide attempt in ulcerative colitis patient after 4 months of infliximab therapy--a case report. J Crohns Colitis. 2010 Nov;4(5):591-3. PMID: 21122565. doi: 10.1016/j.crohns.2010.04.001.

22. Jain A, Marrie RA, Shafer LA, Graff LA, Patten SB, El-Gabalawy R, et al. Incidence of Adverse Psychiatric Events During Treatment of Inflammatory Bowel Disease With Biologic Therapies: A Systematic Review. Crohn's & Colitis 360. 2020;2(1):otz053. doi: 10.1093/crocol/otz053.

23. Figiel I. Pro-inflammatory cytokine TNF-alpha as a neuroprotective agent in the brain. Acta neurobiologiae experimentalis. 2008;68(4):526-34. PMID: 19112477.

24. Courtney MJ, Akerman KE, Coffey ET. Neurotrophins protect cultured cerebellar granule neurons against the early phase of cell death by a two-component mechanism. J Neurosci. 1997 Jun 1;17(11):4201-11. PMID: 9151737.

25. Venters HD, Broussard SR, Zhou JH, Bluthé RM, Freund GG, Johnson RW, et al. Tumor necrosis factor(alpha) and insulin-like growth factor-I in the brain: is the whole greater than the sum of its parts? Journal of neuroimmunology. 2001 Oct 1;119(2):151-65. PMID: 11585617. doi: 10.1016/s0165-5728(01)00388-5.

26. Cousins JP, Harper G. Neurobiochemical changes from Taxol/Neupogen chemotherapy for metastatic breast carcinoma corresponds with suicidal depression. Cancer Letters. 1996 1996/12/20/;110(1):163-7. doi: https://doi.org/10.1016/S0304-3835(96)04486-2.

27. Hasler G, van der Veen JW, Tumonis T, Meyers N, Shen J, Drevets WC. Reduced prefrontal glutamate/glutamine and gamma-aminobutyric acid levels in major depression determined using proton magnetic resonance spectroscopy. Arch Gen Psychiatry. 2007 Feb;64(2):193-200. PMID: 17283286. doi: 10.1001/archpsyc.64.2.193.

28. Klimberg VS, McClellan JL. Glutamine, cancer, and its therapy. Am J Surg. 1996 Nov;172(5):418-24. PMID: WOS:A1996VR81800004.

29. Modi D, Bhalavat R, Patterson JC, 2nd. Suicidal and homicidal behaviors related to dextromethorphan abuse in a middle-aged woman. J Addict Med. 2013 Mar-Apr;7(2):143-4. PMID: 23388679. doi: 10.1097/ADM.0b013e318281a547.

30. Matin N, Kurani A, Kennedy CA, Liu QY. Dextromethorphan-induced near-fatal suicide attempt in a slow metabolizer at cytochrome P450 2D6. The American journal of geriatric pharmacotherapy. 2007 Jun;5(2):162-5. PMID: 17719518. doi: 10.1016/j.amjopharm.2007.05.001.

31. Logan BK, Yeakel JK, Goldfogel G, Frost MP, Sandstrom G, Wickham DJ. Dextromethorphan abuse leading to assault, suicide, or homicide. Journal of forensic sciences. 2012;57(5):1388-94.

32. Modi D, Bhalavat R, Patterson JC. Suicidal and homicidal behaviors related to dextromethorphan abuse in a middle-aged woman. Journal of Addiction Medicine. 2013;7(2):143-4.

33. AUVELITY (dextromethorphan hydrobromide and bupropion hydrochloride) extended-release tablets, for oral use. 2022 [cited 2022 September 20]; Available from: https://www.accessdata.fda.gov/drugsatfda_docs/label/2022/215430s000lblCorrect2.pdf.

34. del Rosario MA, Bender BG, White CW. Suicidal ideation and thought disorder associated with the formoterol component of combined asthma medication. Pediatric Pulmonology. 2013 2013/01/01;48(1):102-3. doi: https://doi.org/10.1002/ppul.22563.

35. del Rosario MA, Bender BG, White CW. Suicidal ideation and thought disorder associated with the formoterol component of combined asthma medication. Pediatr Pulmonol. 2013 Jan;48(1):102-3. PMID: 22489078. doi: 10.1002/ppul.22563.
